# Psychometric evaluation and community norms of the Somatic Symptom Scale-8 based on a representative German sample

**DOI:** 10.3389/fpsyt.2026.1851921

**Published:** 2026-05-22

**Authors:** Sören Kliem, Anna Lohmann, Sebastian Fischer, Markus Zenger, Dirk Baier, Johannes Kruse, Elmar Brähler, Hanna Kampling

**Affiliations:** 1 Department of Social Welfare, Ernst-Abbe University of Applied Sciences Jena, Jena, Germany; 2 Faculty of Applied Human Studies, University of Applied Sciences Magdeburg and Stendal, Stendal, Germany; 3 Integrated Research and Treatment Center Adiposity Diseases - Behavioral Medicine Psychosomatic Medicine and Psychotherapy, University of Leipzig Medical Center, Leipzig, Germany; 4 Institute of Delinquency and Crime Prevention, Zurich University of Applied Sciences, Zurich, Switzerland; 5 CrimeLab, University of Zurich, Zurich, Switzerland; 6 Department of Psychosomatic Medicine and Psychotherapy, Justus Liebig University Giessen, Giessen, Germany; 7 Department for Psychosomatic Medicine and Psychotherapy, Medical Center of the Philipps University Marburg, Marburg, Germany; 8 Department of Psychosomatic Medicine and Psychotherapy, University Medical Center of the Johannes Gutenberg University Mainz, Mainz, Germany; 9 Clinic and Polyclinic for Psychosomatic Medicine and Psychotherapy, Rostock University Medical Center, Rostock, Germany

**Keywords:** measurement invariance, population norms, psychometric evaluation, representative sample, somatic symptoms, SSS-8

## Abstract

**Background:**

The Somatic Symptom Scale-8 (SSS-8) is a brief self-report instrument assessing somatic symptom burden derived from the PHQ-15. The aims of the present study were providing updated population norms for Germany based on a new representative sample and enabling a direct comparison with the original 2012 normative sample. In addition, the study aimed to examine the psychometric properties of the SSS-8, evaluate construct validity, and test factorial validity and measurement invariance.

**Methods:**

A representative German community sample was surveyed in 2021 (*N* = 2,515; 51.6% female; age range: 16–101 years). Construct validity was evaluated through latent correlations with measures of depression (PHQ-2), anxiety (GAD-2), well-being (WHO-5), and loneliness (UCLA-3). Health care utilization was predicted using negative binomial regression. Factorial validity was examined using confirmatory factor analysis with WLSMV estimation (unidimensional model and second-order factor model). Measurement invariance was tested across gender, age, gender × age, depression (PHQ-2 ≥ 3), and anxiety (GAD-2 ≥ 3). Population norms were established using SCAM-smoothed cumulative percentiles.

**Results:**

Internal consistency was high (ω = .922, 95% CI [.915,.929]). The higher-order model (CFI = .979, TLI = .966, SRMR = .046; RMSEA = .091, 90% CI [.083,.099]) showed better fit than the unidimensional model (CFI = .961, TLI = .945, SRMR = .054; RMSEA = .115, 90% CI [.108,.123]). Strict invariance was supported for depression and anxiety. For gender, age, and gender × age, partial scalar invariance was established after freeing one item intercept (headaches). Latent correlations supported convergent and discriminant validity with depression (r = .81), anxiety (r = .76), well-being (r = -.67), and loneliness (r = .57). Each one-point increase in SSS-8 scores was associated with a 9.7% increase in doctor visits (IRR = 1.097, 95% CI [1.082, 1.112]). A cross-cohort comparison with the original normative sample (2012; *N* = 2,484) revealed a significant increase in somatic symptom burden (*d* = -0.40, 95% CI [-0.45, -0.34]).

**Conclusions:**

The SSS-8 demonstrates good psychometric properties in a representative German community sample. Updated population norms are provided for clinical use. The observed increase in somatic symptom burden underscores the necessity of updating population norms at regular intervals.

## 1 Introduction

Somatic symptoms are among the most common reasons for seeking medical care and are associated with substantial health care utilization, functional impairment, and reduced quality of life (1–3). Although somatic symptoms frequently co-occur with depressive and anxiety disorders, they represent a distinct dimension of symptom burden that independently predicts health care utilization even after controlling for depression and anxiety (4, 5). Prevalence estimates suggest that more than one-third of somatic symptoms reported in primary care remain medically unexplained (6, 7), although the field has increasingly moved toward the broader concept of persistent physical symptoms (8), highlighting the need for valid and reliable screening instruments that capture somatic symptom burden in both clinical and epidemiological settings.

The Patient Health Questionnaire-15 (9) has been widely used as a self-report measure of somatic symptom severity. However, its length and the inclusion of gender-specific items have motivated the development of shorter alternatives. The Somatic Symptom Scale-8 (4) was developed as a brief, reliable measure of somatic symptom burden derived from the PHQ-15. The SSS-8 assesses eight common somatic symptoms across four clusters: gastrointestinal problems (1 item), pain (3 items), cardiopulmonary symptoms (2 items), and fatigue (2 items). Items are rated on a five-point Likert scale (0 = not at all to 4 = very much) with a recall period of seven days, yielding a total score of 0-32. In the original validation study using a representative German community sample (*N* = 2,510; data collected in 2012), Gierk et al. (4) reported good internal consistency (α = .81), established severity categories, and demonstrated that SSS-8 scores independently predicted health care utilization after controlling for depression and anxiety. Gierk et al. (10) demonstrated that the SSS-8 exhibits reliability and validity comparable to the PHQ-15 in detecting somatic symptom burden, while being substantially shorter and free of gender-specific items. In a comprehensive systematic review and meta-analysis of 305 studies (*N* = 361,243), Hybelius et al. (11) confirmed that both the PHQ-15 and SSS-8 reflect a combination of domain-specific factors (cardiopulmonary, fatigue, gastrointestinal, pain) and a general somatic symptom burden factor. The pooled internal consistency for the SSS-8 was adequate (*α* = .80, 95% CI [.77,.83]) across 20 studies, and pooled correlations with other measures of somatic symptom burden were high (r = .82, 95% CI [.72,.92]). Expanding its epidemiological utility, Kliem et al. (12) validated the SSS-8 in a large, representative sample of more than 10,000 German adolescents (13–18 years), confirming the original multidimensional factor structure, good internal consistency, measurement invariance across gender and migration background, and meaningful associations with depression, anxiety, social support, health care utilization, and school absenteeism. Within the broader landscape of ultra-brief somatic screening tools, the SSS-8 shares significant conceptual and structural similarities with the brief form of the Giessen Subjective Complaints List [GBB-8 (13),] which comprises four somatic subscales (exhaustion, gastrointestinal, musculoskeletal, cardiovascular complaints) and provides population norms based on a representative German adult sample.

Population norms for screening instruments are not static. Symptom levels in the general population shift over time due to demographic changes, evolving health behaviors, and societal stressors (14–16). For the SSS-8, the original German norms were derived from a representative community sample collected in 2012 (4). Whether these norms still reflect the current symptom burden is unclear, particularly given recent societal events such as the COVID-19 pandemic, which has been associated with increases in somatic symptom reporting (17, 18). Beyond their descriptive function, contemporary norms are required for the calculation of reliable change and clinical significance according to established criteria (19). The primary aim of this study is therefore to provide updated population norms for the SSS-8 based on a new representative German sample. Furthermore, the following aims were pursued: (i) to replicate the factorial validity of the SSS-8 using confirmatory factor analysis, (ii) to replicate measurement invariance (MI) across gender and age and to test MI between groups with elevated and normal to low levels of depression and anxiety symptoms, (iii) to evaluate construct validity through associations with depression, anxiety, well-being, and loneliness, (iv) to replicate the association between SSS-8 scores and health care utilization, and (v) to provide updated population norms and compare them with the original norms from 2012.

## 2 Methods

### 2.1 Procedure

**Figure 1:**
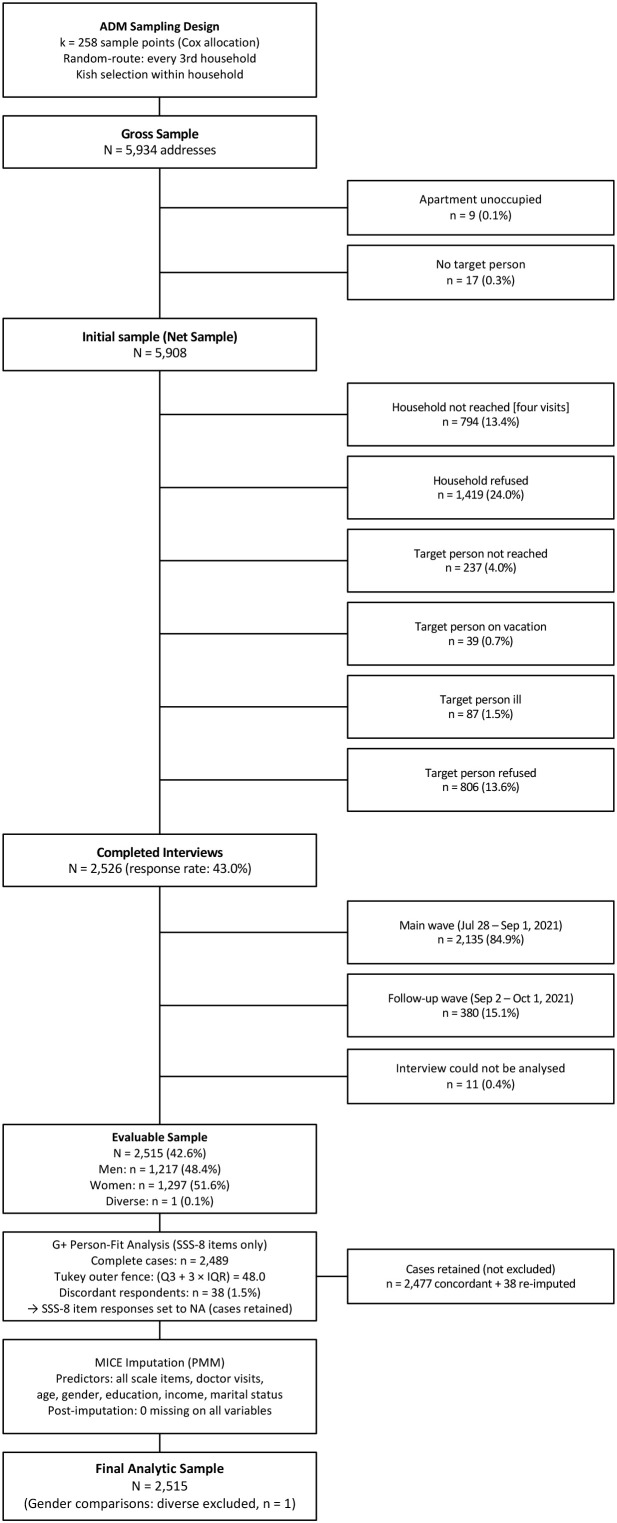
Participant flowchart and data preparation procedure.

Data were collected between July and October 2021 as part of a representative German community survey conducted by a demographic research institute (USUMA GmbH, Berlin). The study was approved by the ethics committee of the Medical Faculty of the University of Leipzig (reference: 298/21-ek). All participants provided written informed consent. The sampling procedure followed the ADM (Arbeitskreis Deutscher Markt- und Sozialforschungsinstitute) face-to-face design. In a first step, 258 sample points were selected using Cox allocation proportional to the population of each federal state. In a second step, households were selected using a random-route procedure with every third household being approached. In a third step, the target person within each household was selected using the Kish method (20). The minimum age for participation was 16 years. The initial gross sample comprised 5,934 addresses, after removing 26 quality-neutral cases (e.g., uninhabited dwellings, no target person in household), the net sample was 5,908. Of these, 2,526 interviews were completed (response rate: 43.0%), of which 2,515 were evaluable (42.6%). Data collection was carried out by 195 trained interviewers in a main wave (July 28 to September 1, 2021; *n* = 2,135, 84.9%) and a follow-up wave (September 2 to October 1, 2021; *n* = 380, 15.1%). No restrictive COVID-19 regulations were in effect during the field period. **Figure 1** provides an overview of the sampling and data preparation procedure. Data collection comprised two parts: (a) an interviewer-administered section covering sociodemographic characteristics following the standards of the German Federal Statistical Office (Statistisches Bundesamt), and (b) a paper-based self-report questionnaire containing the psychological instruments. Interviewers remained available for questions but did not interfere with responses.

### 2.2 Sample

Data from *N* = 2,515 participants (51.6% female; mean age: 50.1 years, SD = 18.0, range: 16–101) were analyzed. One participant identifying as diverse was excluded from gender-specific analyzes. Detailed sample characteristics are provided in **Table 1**, and an overview of the sampling and data preparation procedure is shown in **Figure 1**.

**Table 1:** Demographic characteristics of the study sample.

Characteristic	Total sample (*N* = 2,515)	Sample of men (*N* = 1,217)	Sample of women (*N* = 1,297)
Age (Years)			
Mean (SD)	50.1 (18.0)	49.5 (18.2)	50.6 (17.9)
Median [Min, Max]	51.0 [16.0, 101]	51.0 [16.0, 101]	51.0 [16.0, 93.0]
Age Groups			
16-24	225 (8.9%)	124 (10.2%)	101 (7.8%)
25-34	389 (15.5%)	199 (16.4%)	190 (14.6%)
35-44	367 (14.6%)	148 (12.2%)	219 (16.9%)
45-54	433 (17.2%)	204 (16.8%)	229 (17.7%)
55-64	487 (19.4%)	266 (21.9%)	221 (17.0%)
65-74	376 (15.0%)	177 (14.5%)	199 (15.3%)
75+	238 (9.5%)	99 (8.1%)	138 (10.6%)
Marital Status			
Married/living together	1001 (39.8%)	511 (42.0%)	490 (37.8%)
Married/separated	44 (1.7%)	24 (2.0%)	20 (1.5%)
Single	797 (31.7%)	448 (36.8%)	349 (26.9%)
Divorced	404 (16.1%)	170 (14.0%)	234 (18.0%)
Widowed	265 (10.5%)	62 (5.1%)	202 (15.6%)
Missing	4 (0.2%)	2 (0.2%)	2 (0.2%)
Living with Partner			
No	1170 (46.5%)	528 (43.4%)	641 (49.4%)
Yes	1317 (52.4%)	675 (55.5%)	642 (49.5%)
Missing	28 (1.1%)	14 (1.2%)	14 (1.1%)
Education Level			
Low (ISCED 0-2)	728 (28.9%)	361 (29.7%)	366 (28.2%)
Medium (ISCED 3-4)	1482 (58.9%)	686 (56.4%)	796 (61.4%)
High (ISCED 5+)	256 (10.2%)	142 (11.7%)	114 (8.8%)
Other	46 (1.8%)	28 (2.3%)	18 (1.4%)
Missing	3 (0.1%)	0 (0%)	3 (0.2%)
Employment Status			
Full-time employed	1127 (44.8%)	683 (56.1%)	444 (34.2%)
Part-time employed	353 (14.0%)	50 (4.1%)	303 (23.4%)
In education/training	172 (6.8%)	97 (8.0%)	75 (5.8%)
Unemployed	111 (4.4%)	72 (5.9%)	39 (3.0%)
Retired	668 (26.6%)	308 (25.3%)	359 (27.7%)
Other	76 (3.0%)	2 (0.2%)	74 (5.7%)
Missing	8 (0.3%)	5 (0.4%)	3 (0.2%)
Nationality			
German	2410 (95.8%)	1166 (95.8%)	1243 (95.8%)
Not German	105 (4.2%)	51 (4.2%)	54 (4.2%)
Household Size			
1	995 (39.6%)	468 (38.5%)	526 (40.6%)
2	891 (35.4%)	465 (38.2%)	426 (32.8%)
3	335 (13.3%)	154 (12.7%)	181 (14.0%)
4+	294 (11.7%)	130 (10.7%)	164 (12.6%)
Children < 16 in Household			
No	2100 (83.5%)	1059 (87.0%)	1040 (80.2%)
Yes	415 (16.5%)	158 (13.0%)	257 (19.8%)
Per Capita Income (EUR)			
Mean (SD)	1520 (761)	1570 (804)	1460 (714)
Median [Min, Max]	1420 [62.5, 6250]	1420 [250, 6250]	1380 [62.5, 6250]
Missing	45 (1.8%)	16 (1.3%)	29 (2.2%)

Education levels based on ISCED-2011: Low, no degree or lower secondary (ISCED 0-2); Medium, upper secondary to post-secondary non-tertiary (ISCED 3-4); High, tertiary (ISCED 5+); Other, other degree or currently in school. Employment: Full-time, >= 35 h/week; Part-time, < 35 h/week; Other includes parental leave and homemakers. Per capita income derived from midpoints of 13 household income categories divided by household size. One participant identified as diverse, included in the Total Sample but excluded from gender-stratified analyzes.

### 2.3 Instruments

#### 2.3.1 The Somatic Symptom Scale-8

The Somatic Symptom Scale-8 [SSS-8 (4),] is a brief self-report questionnaire assessing somatic symptom burden during the past seven days. The SSS-8 comprises eight items covering four symptom clusters: gastrointestinal (1 item), pain (3 items), cardiopulmonary (2 items), and fatigue (2 items). Items are rated on a five-point Likert scale ranging from 0 (not at all) to 4 (very much), yielding a total score between 0 and 32, with higher scores indicating greater somatic symptom burden. Severity categories have been proposed as follows: no to minimal (0–3), low (4–7), medium (8–11), high (12–15), and very high (16–32). In the original validation study, internal consistency was alpha = .81 (4).

#### 2.3.2 The patient health questionnaire-4

The patient health questionnaire-4 [PHQ-4 (21, 22),] assess symptoms of depression (PHQ-2) and generalized anxiety disorder (GAD-2) on subscales of two items each. Each item is rated on a four-point scale (0 = not at all to 3 = nearly every day), resulting in subscale scores ranging from 0 to 6. Higher scores indicate greater symptom severity. A cut-off of >= 3 indicates clinically relevant depressive (23) or anxiety (24) symptoms. Internal consistency for the PHQ-2, and GAD-2 in this study was acceptable to good [PHQ-2: α = .82, *ω* = .82; GAD-2: α = .76, ω = .77; see Kliem et al. (15) for details].

#### 2.3.3 The World Health Organization-five well-being index

The World Health Organization-Five Well-Being Index [WHO-5 (25), German version (26):] consists of five positively phrased items assessing well-being over the past 14 days. Participants rate each statement on a scale from 0 (at no time) to 5 (all of the time), yielding a total score range of 0–25. Scores are conventionally converted to a percentage scale ranging from 0 (lowest well-being) to 100 (highest well-being) by multiplying the raw score by four (25). Internal consistency in the recent study was excellent (α = .95, ω = .95; see Kliem et al. (15) for details).

#### 2.3.4 UCLA loneliness scale (short form)

Loneliness was measured using a three-item version of the UCLA Loneliness Scale [ (27); German version (28):]. Participants rated items on a five-point Likert scale (0 = never to 4 = very often), with higher scores indicating greater loneliness. The total score ranges from 0 to 12. The German version demonstrated very good reliability in this study [α = .90, ω = .96; see Kliem et al. (15)].

#### 2.3.5 Health care utilization

Health care utilization was assessed by the self-reported number of doctor visits in the past three months.

### 2.4 Statistical analysis

#### 2.4.1 Missing data and inconsistent response patterns

Missing values on the SSS-8 items ranged from 0.0% to 0.4% per item. In order to address missing data, we utilized chained equation modeling as outlined in van Buuren and Groothuis-Oudshoorn (29). The imputation model included all scale items (SSS-8, GAD-2, PHQ-2, WHO-5, UCLA-3), health care utilization (doctor visits), age, gender, education, household income, and marital status as predictors. To avoid nonexisting item values, the estimated values (ŷ) were corrected by predictive mean matching (i.e., the observed values closest to the predicted value were chosen). Imputation procedures were implemented using the R package mice (29). Data analysis was carried out on one imputed data set. Inconsistent response patterns (i.e., careless response patterns) were identified using the item-pair-based G+ person-fit statistic (30). G+ scores were computed for all complete cases (*n* = 2,489). Respondents with G+ values exceeding Tukey’s outer fence [ (31); see (32)] were classified as discordant (*n* = 38, 1.5%). Rather than excluding these cases, their item responses were set to missing and subsequently re-estimated via multiple imputation, thereby preserving the full sample size.

Mean and standard deviations were obtained for all SSS-8 items in the total sample as well as for the sub samples of men and women. Group differences between men and women were tested using permutation tests with 10,000 resamples (33). Effect sizes were quantified using Cliff’s δ with 95% confidence intervals (34) for individual items (ordinal data) and Cohen’s d with 95% confidence intervals for the SSS-8 total score (35). To assess item characteristics we examined the skewness and kurtosis, item difficulty (i.e. percentage of participants endorsing each item), item-total correlations (rit), and Cronbach’s alpha if the item was deleted. Item characteristics were obtained using the R package psych (36).

#### 2.4.2 Confirmatory factor analysis

Factorial validity was examined using confirmatory factor analysis (CFA) with the lavaan package (37). Given the ordered categorical nature of the items, weighted least squares mean and variance adjusted (WLSMV) estimation was used (38). Two models were compared: a unidimensional model (M1) and a higher-order model reflecting the four symptom clusters proposed by Gierk et al. [M2 (4)]. In the higher-order model, a second-order general somatic symptom burden factor explains the covariance among three first-order domain factors: pain (Items 2–4: back pain, limb pain, headaches), cardiopulmonary symptoms (Items 5–6: chest pain/shortness of breath, dizziness), and fatigue (Items 7–8: feeling tired/low energy, trouble sleeping). In M2, the gastrointestinal item (Item 1) was loaded directly onto the higher-order factor because specifying a single-item first-order factor resulted in a non-invertible information matrix under WLSMV estimation. To evaluate goodness-of-fit of the relevant model, the following four criteria were considered. While the RMSEA and the 90% confidence interval both assess absolute model fit, the two additionally calculated criteria (Comparative Fit Index [CFI] and Tucker Lewis Index [TLI]) measure relative model fit compared to the “null” model. RMSEA values < 0.050 represent a close fit, values between 0.050 and 0.080 represent a reasonably close fit, and values > 0.080 represent an unacceptable model (39). Regarding CFI and TLI, Hu and Bentler (39) suggested a CFI and TLI > 0.900 for an adequate fit and a CFI and TLI > 0.950 for a good model fit. In addition, the Standardized Root Mean Square Residual (SRMR) was considered as an indicator of the average standardized residuals between observed and predicted covariances; SRMR values < 0.080 are generally interpreted as indicative of good fit (39). Because WLSMV estimation applies a mean and variance correction to the chi-square statistic, all fit indices reported in this study are the scaled variants (i.e., robust CFI, robust TLI, robust RMSEA with 90% CI). SRMR does not have a scaled variant under WLSMV and is reported as the standard estimate. Model comparison was performed using the scaled chi-square difference test (Satorra–Bentler method).

#### 2.4.3 Reliability

To account for potential issues arising from unmet assumptions in the calculation of coefficient alpha (40), we assessed internal consistency using McDonald’s omega, computed from standardized CFA factor loadings (41) of M1 (see **Supplementary eFigure 2**). 95% confidence intervals for omega and alpha were obtained via bootstrap with 1,000 resamples.

#### 2.4.4 Measurement invariance

Measurement invariance (MI) was tested using multiple group confirmatory factor analysis (MGCFA) following the procedure suggested by Wu and Estabrook (42) for ordered categorical data. The higher-order model (M2) was used with theta parameterization. Five nested models were tested: (i) configural invariance (unconstrained except for identification constraints), (ii) threshold invariance (equal thresholds across groups), (iii) weak invariance (equal factor loadings), (iv) strong invariance (equal intercepts), and (v) full invariance (equal residual variances). The parameter constraints for each model are visualized in **Supplementary eFigure 1**. Chen’s cut-off criteria were used (43), with a change of < -.010 in CFI and a change of >= .015 in RMSEA indicating non-invariance. MI was tested across five grouping variables: gender (male vs. female), age (below vs. above median), gender × age (four groups), clinical status defined by PHQ-2 >= 3 (24) as well as GAD-2 >= 3 (25). Sparse response categories were collapsed prior to analysis (categories 3 and 4 were merged) to ensure model identification across all subgroups. The *n* = 1 participant identifying as diverse was excluded from gender-specific comparisons due to insufficient subgroup size.

#### 2.4.5 Construct validity

Construct validity was evaluated through latent correlations estimated in structural equation models with WLSMV estimation. 95% confidence intervals for latent correlations were obtained via bootstrap with 1,000 resamples. The following hypotheses were formulated: somatic symptom levels should be (a) positively associated with depression (PHQ-2) and anxiety (GAD-2), supporting convergent validity, (b) negatively associated with well-being (WHO-5), supporting divergent validity, and (c) more weakly associated with loneliness (UCLA-3) than with depression and anxiety, supporting discriminant validity. The association between SSS-8 scores and health care utilization was examined using negative binomial regression (44), controlling for depression (PHQ-2), anxiety (GAD-2), age, and gender. Two models were estimated: Model 1 with the SSS-8 total score as a continuous predictor, and Model 2 with SSS-8 severity categories (4) as a categorical predictor. Incidence rate ratios (IRR) with 95% confidence intervals are reported.

#### 2.4.6 Population norms

Population norms were established using SCAM-smoothed cumulative percentiles (45). For each possible sum score, the proportion of participants scoring at or below that value was computed. To ensure monotonicity and reduce sampling noise, a shape-constrained additive model (SCAM) with monotonically increasing P-splines was fitted to the raw cumulative percentages. 95% confidence intervals were obtained from 1,000 bootstrap resamples for the total sample and gender-specific norms. Age-specific norms are reported as point estimates without confidence intervals due to limited subgroup sizes.

#### 2.4.7 Cross-cohort comparison

To examine temporal changes in somatic symptom burden, item-level and total score comparisons were conducted between the present sample and the normative sample collected by Gierk et al. (4) in 2012 (*N* = 2,484 after excluding participants aged 14–15 to match the minimum age of 16 in the present sample). Effect sizes were quantified as Cliff’s δ with 95% confidence intervals for individual items (ordinal scale, values range from –1 to +1, with values close to 0 indicating negligible group differences) and Cohen’s d with 95% confidence intervals for the total score (35). Distributional shifts in severity categories were tested using Pearson’s chi-square test, with Cramér’s V as effect size measure. Outlier detection and imputation were applied to the 2012 data using the same procedures as for the main sample.

## 3 Results

### 3.1 Item characteristics

The mean SSS-8 total score was M = 4.64 (SD = 4.86). The distribution of severity categories was as follows: no to minimal 53.8%, low 24.6%, medium 11.8%, high 5.6%, and very high 4.3%. **Table 2** presents the item-level descriptive statistics. Item means ranged from 0.23 (Item 5: chest pain or shortness of breath) to 0.91 (Item 2: back pain). Item difficulty indices (Pi) ranged from 5.70% (Item 5) to 22.84% (Item 2). Corrected item-total correlations ranged from .49 (Item 4: headaches) to .74 (Item 7: feeling tired or having low energy), indicating adequate to good item discrimination. Inter-item correlations ranged from .29 (Items 3 and 4) to .68 (Items 7 and 8; see **Supplementary Table 5** in **Supplementary Material**). Cronbach’s alpha if item deleted ranged from .83 to .86, suggesting that no single item removal would meaningfully increase internal consistency. Standardized factor loadings from the unidimensional CFA (see 3.2) ranged from .57 (Item 4: headaches) to .86 (Item 7: feeling tired or having low energy). **Supplementary eTable 1** presents the gender comparisons. Women reported significantly higher SSS-8 total scores (M = 5.14, SD = 5.03) than men (M = 4.10, SD = 4.61), corresponding to a small effect [d = -0.22, 95% CI (-0.29, -0.14), permutation p <.001]. At the item level, the largest gender differences were observed for headaches [δ = -0.24, 95% CI (-0.27, -0.19)] and gastrointestinal problems [δ = -0.15, 95% CI (-0.18, -0.11)], whereas back pain [δ = 0.01, 95% CI (-0.04, 0.05)] and limb pain [δ = -0.01, 95% CI (-0.05, 0.03)] showed negligible differences.

**Table 2:** Item statistics with descriptive and psychometric indices SSS-8 (*N* = 2,515).

SSS-8 item	*M*	*SD*	*Skew*	*Kurt*	*Pi*	*r_it_*	*α^-1^*	*λ*
Stomach or bowel problems	0.46	0.73	1.58	1.89	11.39	.55	.85	.66
Back pain	0.91	0.98	0.84	-0.09	22.84	.61	.85	.76
Pain in arms/legs/joints	0.67	0.95	1.33	0.92	16.68	.65	.84	.81
Headaches	0.71	0.81	1.03	0.76	17.68	.49	.86	.57
Chest pain/ shortness of breath	0.23	0.61	3.03	9.55	5.70	.61	.85	.84
Dizziness	0.27	0.65	2.89	9.17	6.67	.62	.85	.83
Feeling tired/ low energy	0.75	1.00	1.25	0.75	18.82	.74	.83	.86
Trouble sleeping	0.65	0.96	1.50	1.58	16.14	.69	.84	.82
*SSS-8 Total*	*4.64*	*4.86*	*1.55*	*2.60*				

M, Mean; SD, Standard Deviation; Skew, skewness; Kurt, excess kurtosis; Pi, item difficulty index (% of max score); r_it_, corrected item-total correlation; α^-1^, Cronbach’s alpha if item deleted; λ, standardized factor loading from unidimensional CFA (M1, WLSMV).

### 3.2 Factorial validity

The unidimensional model (M1) showed the following fit: χ^2^ (20) = 688, CFI = .961, TLI = .945, RMSEA = .115 [90% CI (.108, .123)], SRMR = .054 The higher-order model (M2) showed improved fit relative to M1: χ^2^ (17) = 373, CFI = .979, TLI = .966, RMSEA = .091 (90% CI [.083, .099]), SRMR = .046. The scaled chi-square difference test supported M2 over M1 (χ^2^ difference = 260, df = 3, p <.001). RMSEA exceeded the conventional threshold of .080 in both models, including M2. This pattern is consistent with the known inflation of RMSEA for models with few items and large sample sizes under WLSMV estimation (46), but should not be dismissed on this basis alone. We therefore evaluated model fit across multiple indices: CFI and TLI for M2 exceeded the .950 threshold for good fit, and SRMR was below the .080 threshold in both models. All standardized factor loadings in M2 exceeded the minimum threshold of .40. At the item level, loadings ranged from .61 (Item 4: headaches on the pain factor) to .90 (Item 7: feeling tired on the fatigue factor). The second-order loadings indicated that all symptom clusters were well represented by the higher-order somatic symptom factor (see **Supplementary eFigure 3**).

### 3.3 Reliability

McDonald’s omega of the SSS-8 for the full sample was ω = .922, 95% CI (.915, .929). Cronbach’s alpha of the SSS-8 for the full sample was α = .863, 95% CI (.851, .875). Gender-stratified estimates were: men ω = .926, 95% CI (.916, .935), α = .859, 95% CI (.840, .875); women ω = .919, 95% CI (.910, .928), α = .866, 95% CI (.851, .880). Both estimates exceeded the value of α = .81 reported by Gierk et al. (4).

### 3.4 Measurement invariance

**Supplementary eTable 2** (see **Supplementary Material**) presents the measurement invariance results for all five grouping variables. The transition from configural to threshold invariance showed positive changes in CFI across all groupings, which is expected under theta parameterization for ordered categorical data. Metric invariance was supported across all grouping variables (all ΔCFI ≥ −.010). Full scalar invariance was supported for depression (PHQ-2 ≥ 3) and anxiety (GAD-2 ≥ 3), and strict invariance was established for these two grouping variables.

For gender, age (median split), and gender × age (four groups), the transition from metric to scalar invariance exceeded the Chen threshold (44) (gender: ΔCFI = −.011; age: ΔCFI = −.025; gender × age: ΔCFI = −.034). A one-at-a-time analysis, in which only one intercept constraint was freed at each step, identified Item 4 (headaches) as the primary source of non-invariance across all three groupings. After freeing the intercept constraint for Item 4, partial scalar invariance was established for all three groupings (gender: ΔCFI = −.003, ΔRMSEA = +.002; age: ΔCFI = −.005, ΔRMSEA = +.003; gender × age: ΔCFI = −.010, ΔRMSEA = +.008; all relative to the metric model). In the full invariance sequence, the transition from scalar to strict invariance showed negligible changes in CFI for these groupings (all ΔCFI ≥ −.004), indicating that residual variances were equivalent across groups.

### 3.5 Construct validity

**Supplementary eTable 3** (see **Supplementary Material**) presents the latent and manifest correlations between the SSS-8 and the validation instruments. Supporting convergent validity, the SSS-8 showed substantial positive latent correlations with depression [PHQ-2: r = .80, 95% CI (.77,.83)] and anxiety [GAD-2: r = .76, 95% CI (.72,.79)]. Supporting divergent validity, a substantial negative latent correlation with well-being was observed [WHO-5: r = -.67, 95% CI (-.70, -.64)]. The latent correlation with loneliness [UCLA-3: r = .57, 95% CI (.53,.61)] was weaker than the convergent correlations, consistent with the expectation that loneliness and somatic symptoms are related but distinct constructs, supporting discriminant validity. The manifest correlations followed the same pattern but were lower in magnitude, as expected given that latent correlations correct for measurement error: SSS-8 x PHQ-2: r = .652 [95% CI (.629,.674)]; SSS-8 x GAD-2: r = .596 [95% CI (.570,.621)]; SSS-8 x WHO-5: r = -.596 [95% CI (-.621, -.570)]; SSS-8 x UCLA-3: r = .486 [95% CI (.455,.515)].

Participants reported a mean of 1.51 doctor visits (SD = 2.74, Mdn = 1) in the past three months. The overdispersion parameter (theta = 1.35) confirmed the use of negative binomial regression. Even after controlling for depression (PHQ-2), anxiety (GAD-2), age, and gender, each one-point increase in the SSS-8 total score was associated with a 9.7% increase in doctor visits [IRR = 1.097, 95% CI (1.082, 1.112), p <.001]. Neither GAD-2 [IRR = 1.015, 95% CI (0.956, 1.078), p = .62] nor PHQ-2 scores [IRR = 0.960, 95% CI (0.904, 1.020), p = .19] were significant predictors after accounting for the SSS-8, age, and gender. Age [IRR = 1.011, 95% CI (1.008, 1.013), p <.001] and female gender [IRR = 1.321, 95% CI (1.197, 1.459), p <.001] were additional significant predictors. When severity categories were used as the predictor, mean doctor visits increased monotonically across severity groups: no to minimal = 0.86 (SD = 1.33), low = 1.57 (SD = 1.97), medium = 2.60 (SD = 5.69), high = 2.99 (SD = 2.83), and very high = 4.29 (SD = 3.58). These results closely replicate the findings of Gierk et al. (4), who reported an IRR of 1.12 per point and 1.53 per severity level, noting that the present study assessed doctor visits over three months rather than twelve months.

### 3.6 Population norms

**Table 3** presents the smoothed cumulative percentile norms for the SSS-8 total score in the total sample and separately for men and women. The full age- and gender-specific norms are provided in **Supplementary Data Sheet 1**, covering all SSS-8 sum scores from 0 to 32 across seven age groups (< 25, 25–34, 35–44, 45–54, 55–64, 65–74, ≥ 75 years). For the total sample (*N* = 2,515), a score of 0 corresponded to the 18.7^th^ percentile [95% CI (17.1, 20.3)], a score of 4 to the 61.3^rd^ percentile [95% CI (59.5, 62.9)], a score of 8 to the 82.4^th^ percentile [95% CI (80.9, 83.8)], and a score of 12 to the 91.9^th^ percentile [95% CI (90.8, 92.8)]. Gender-specific norms indicated that men scored lower on average: a score of 4 corresponded to the 66.2^nd^ percentile in men [95% CI (63.7, 68.6)] but only the 56.8^th^ percentile in women [95% CI (54.4, 59.4)].

**Table 3:** Smoothed cumulative percentiles for the SSS-8 sum score for the total sample and by gender.

SSS-8	Total sample (*N* = 2,515)	Sample of men (*N* = 1,217)	Sample of women (*N* = 1,297)
0	18.7 [17.1; 20.3]	22.6 [20.2; 24.9]	15.1 [13.2; 17.2]
1	31.6 [30.0; 33.1]	36.5 [34.0; 39.0]	27.2 [24.9; 29.5]
2	43.0 [41.2; 44.7]	48.2 [45.7; 50.9]	38.2 [35.8; 40.9]
3	52.9 [51.1; 54.5]	58.1 [55.6; 60.6]	48.1 [45.7; 50.9]
4	61.3 [59.5; 62.9]	66.2 [63.7; 68.6]	56.8 [54.4; 59.4]
5	68.3 [66.5; 69.9]	72.8 [70.4; 75.1]	64.2 [61.8; 66.9]
6	74.1 [72.2; 75.6]	78.1 [75.7; 80.3]	70.3 [68.0; 72.8]
7	78.7 [77.0; 80.2]	82.4 [80.2; 84.4]	75.2 [73.1; 77.5]
8	82.4 [80.9; 83.8]	85.7 [83.8; 87.5]	79.3 [77.3; 81.5]
9	85.5 [84.0; 86.7]	88.4 [86.6; 89.9]	82.7 [80.8; 84.8]
10	88.0 [86.6; 89.1]	90.5 [88.8; 92.0]	85.6 [83.9; 87.6]
11	90.1 [88.9; 91.2]	92.3 [90.7; 93.6]	88.1 [86.5; 89.8]
12	91.9 [90.8; 92.8]	93.7 [92.3; 94.9]	90.2 [88.8; 91.7]
13	93.3 [92.4; 94.2]	94.8 [93.5; 95.9]	92.0 [90.7; 93.3]
14	94.6 [93.7; 95.4]	95.7 [94.5; 96.7]	93.6 [92.3; 94.8]
15	95.6 [94.8; 96.4]	96.4 [95.4; 97.4]	94.9 [93.8; 96.0]
16	96.5 [95.8; 97.2]	97.0 [96.1; 97.9]	96.1 [95.1; 97.0]
17	97.3 [96.6; 97.8]	97.5 [96.7; 98.4]	97.0 [96.2; 97.8]
18	97.9 [97.3; 98.4]	98.0 [97.2; 98.7]	97.8 [97.0; 98.4]
19	98.4 [97.9; 98.8]	98.4 [97.7; 99.1]	98.4 [97.7; 98.9]
20	98.8 [98.4; 99.1]	98.8 [98.2; 99.3]	98.8 [98.2; 99.3]
21	99.1 [98.8; 99.4]	99.2 [98.7; 99.5]	99.1 [98.6; 99.5]
22	99.4 [99.1; 99.6]	99.5 [99.1; 99.7]	99.3 [98.9; 99.7]
23	99.6 [99.3; 99.8]	99.7 [99.4; 99.8]	99.5 [99.1; 99.8]
24	99.7 [99.5; 99.9]	99.9 [99.6; 99.9]	99.6 [99.3; 99.9]
25	99.8 [99.6; >99.9]	99.9 [99.7; >99.9]	99.7 [99.4; >99.9]
26	99.9 [99.7; >99.9]	>99.9 [99.8; >99.9]	99.8 [99.5; >99.9]
27	99.9 [99.8; >99.9]	>99.9 [99.9; >99.9]	99.8 [99.6; >99.9]
28	99.9 [99.8; >99.9]	>99.9 [>99.9; >99.9]	99.9 [99.7; >99.9]
29	>99.9 [99.9; >99.9]	>99.9 [>99.9; >99.9]	99.9 [99.7; >99.9]
30	>99.9 [99.9; >99.9]	>99.9 [>99.9; >99.9]	>99.9 [99.8; >99.9]
31	>99.9 [99.9; >99.9]	>99.9 [>99.9; >99.9]	>99.9 [99.9; >99.9]
32	>99.9 [99.9; >99.9]	>99.9 [>99.9; >99.9]	>99.9 [99.9; >99.9]

Smoothed via SCAM; 95% CI from 1,000 bootstrap resamples.

### 3.7 Cross-cohort comparison

**Table 4** presents the cross-cohort comparison between the present sample (2021; *N* = 2,515) and the original normative sample (REP 20, 2012; *N* = 2,484) for the total sample; the corresponding gender-stratified comparisons are shown in **Tables 4A** and **4B** for men and women, respectively. The SSS-8 total score was significantly higher in 2021 (M = 4.64, SD = 4.86) than in 2012 (M = 2.93, SD = 3.59), corresponding to a small to medium effect (d = -0.40, 95% CI [-0.45, -0.34]). All eight items showed significantly higher endorsement in 2021 (all p <.001), with effect sizes ranging from δ = -0.05 (95% CI [-0.07, -0.04]) for Item 5 (chest pain or shortness of breath) to δ = -0.19 [95% CI (-0.22, -0.17)] for Item 1 (gastrointestinal problems). The increase was more pronounced in women [d = -0.44, 95% CI (-0.51, -0.36)] than in men [d = -0.37, 95% CI (-0.45, -0.28)]. The distribution of severity categories shifted significantly toward higher severity in 2021 (χ² (4) = 164.58, p <.001, Cramér’s V = .181). As shown in **Supplementary eTable 4** (see **Supplementary Material**), the proportion of participants in the “no to minimal” category decreased from 67.8% (2012) to 53.8% (2021), while the proportions in all higher categories increased (low: 22.5% to 24.6%; medium: 6.4% to 11.8%; high: 2.0% to 5.6%; very high: 1.3% to 4.3%). This shift was observed in both men [χ² (4) = 64.51, p <.001, V = .165] and women [χ² (4) = 104.41, p <.001, V = .200].

**Table 4:** Cross-cohort comparison: SSS-8 item and total scores in 2012 and 2021 for the total sample.

SSS-8 item	2012 total sample (*N* = 2,484)				2021 total sample (*N = 2,515*)				Group differences		
	*M*	*SD*	*Skew*	*Kurt*	*M*	*SD*	*Skew*	*Kurt*	*p*	ES (δ/d)	95% CI
Stomach or bowel problems	0.19	0.50	3.05	9.84	0.46	0.73	1.58	1.89	<.001	δ = -0.19	[-0.22, -0.17]
Back pain	0.68	0.91	1.17	0.47	0.91	0.98	0.84	-0.09	<.001	δ = -0.14	[-0.17, -0.11]
Pain in arms/legs/joints	0.47	0.82	1.76	2.50	0.67	0.95	1.33	0.92	<.001	δ = -0.11	[-0.14, -0.08]
Headaches	0.50	0.71	1.34	1.38	0.71	0.81	1.03	0.76	<.001	δ = -0.15	[-0.17, -0.12]
Chest pain/ shortness of breath	0.14	0.47	3.87	16.33	0.23	0.61	3.03	9.55	<.001	δ = -0.05	[-0.07, -0.04]
Dizziness	0.13	0.42	3.68	14.78	0.27	0.65	2.89	9.17	<.001	δ = -0.08	[-0.10, -0.06]
Feeling tired/ low energy	0.47	0.76	1.76	3.17	0.75	1.00	1.25	0.75	<.001	δ = -0.14	[-0.17, -0.11]
Trouble sleeping	0.37	0.66	2.00	4.43	0.65	0.96	1.50	1.58	<.001	δ = -0.13	[-0.16, -0.11]
*SSS-8 Total*	2.93	3.59	2.06	5.94	4.64	4.86	1.55	2.60	<.001	d = -0.4	[-0.45, -0.34]

**Table 4a:** Cross-cohort comparison: SSS-8 item and total scores in 2012 and 2021 for the sample of men.

SSS-8 item	*2012 n = 1,158*				*2021 n = 1,217*						
	*M*	*SD*	*Skew*	*Kurt*	*M*	*SD*	*Skew*	*Kurt*	*p*	ES (δ/d)	95% CI
Stomach or bowel problems	0.13	0.43	3.7	15.31	0.35	0.67	2.00	3.61	<.001	δ = -0.15	[-0.18, -0.12]
Back pain	0.65	0.91	1.25	0.72	0.92	0.99	0.80	-0.25	<.001	δ = -0.16	[-0.20, -0.12]
Pain in arms/legs/joints	0.44	0.80	1.88	3	0.66	0.95	1.35	0.98	<.001	δ = -0.12	[-0.16, -0.08]
Headaches	0.39	0.66	1.7	2.7	0.54	0.74	1.37	1.60	<.001	δ = -0.10	[-0.14, -0.06]
Chest pain/ shortness of breath	0.14	0.47	3.86	16.32	0.21	0.58	3.13	10.05	.001	δ = -0.04	[-0.07, -0.02]
Dizziness	0.10	0.36	4.45	22.7	0.21	0.56	3.24	12.23	<.001	δ = -0.08	[-0.10, -0.05]
Feeling tired/ low energy	0.42	0.73	2	4.42	0.66	0.95	1.43	1.41	<.001	δ = -0.13	[-0.17, -0.09]
Trouble sleeping	0.33	0.65	2.38	6.67	0.55	0.91	1.74	2.45	<.001	δ = -0.11	[-0.14, -0.07]
*SSS-8 Total*	2.61	3.46	2.4	8.62	4.1	4.61	1.71	3.16	<.001	d = -0.37	[-0.45, -0.28]

M, Mean; SD, Standard Deviation; Skew, skewness; Kurt, kurtosis; Cliff’s δ (ordinal); Total, Cohen’s d (quasi-metric, SSS-8 total score); Negative values, higher scores in 2021.

**Table 4b:** Cross-cohort comparison: SSS-8 item and total scores in 2012 and 2021 for the sample of women.

SSS-8 item	*2012 n = 1,326*				*2021 n = 1,297*						
	*M*	*SD*	*Skew*	*Kurt*	*M*	*SD*	*Skew*	*Kurt*	*p*	ES (δ/d)	95% CI
Stomach or bowel problems	0.23	0.55	2.64	7.08	0.55	0.78	1.28	0.95	<.001	δ = -0.23	[-0.26, -0.20]
Back pain	0.69	0.91	1.1	0.26	0.91	0.98	0.88	0.06	<.001	δ = -0.13	[-0.17, -0.09]
Pain in arms/legs/joints	0.50	0.84	1.66	2.12	0.68	0.96	1.31	0.87	<.001	δ = -0.1	[-0.14, -0.06]
Headaches	0.58	0.73	1.1	0.69	0.87	0.83	0.80	0.43	<.001	δ = -0.2	[-0.23, -0.16]
Chest pain/ shortness of breath	0.14	0.47	3.87	16.32	0.24	0.63	2.94	9.03	<.001	δ = -0.07	[-0.09, -0.04]
Dizziness	0.16	0.46	3.22	10.84	0.32	0.72	2.60	7.06	<.001	δ = -0.09	[-0.12, -0.06]
Feeling tired/ low energy	0.51	0.77	1.58	2.32	0.84	1.04	1.10	0.29	<.001	δ = -0.16	[-0.20, -0.12]
Trouble sleeping	0.40	0.67	1.7	2.82	0.73	0.99	1.32	1.01	<.001	δ = -0.16	[-0.20, -0.12]
*SSS-8 Total*	3.22	3.67	1.81	4.24	5.14	5.03	1.42	2.20	<.001	d = -0.44	[-0.51, -0.36]

M, Mean; SD, Standard Deviation; Skew, skewness; Kurt, kurtosis; Cliff’s δ (ordinal); Total, Cohen’s d (quasi-metric, SSS-8 total score); Negative values, higher scores in 2021.

## 4 Discussion

The present study examined the psychometric properties of the SSS-8 and provided updated population norms based on a representative German community sample collected in 2021. The results support the reliability, factorial validity, construct validity, and measurement invariance of the SSS-8. The higher-order model reflecting the four symptom clusters proposed by Gierk et al. (4) showed better fit than the unidimensional model, supporting the multidimensional structure of the SSS-8. This finding is consistent with meta-analytic evidence supporting a hierarchical structure for the SSS-8 (11, 47). However, the average variance extracted for the general factor ranged from only .30 to .51 across studies (11), indicating that domain-specific factors account for a substantial portion of the variance. Given the dimensional nature of somatic symptom burden (48, 49), clinicians should complement the SSS-8 sum score with a review of the symptom domains that dominate the individual presentation. RMSEA values exceeded conventional thresholds in both models. While this pattern is consistent with the known inflation of RMSEA for short scales with few degrees of freedom and large samples under WLSMV estimation (46), the elevated values should be interpreted with caution. The remaining fit indices (CFI, TLI, SRMR) were within acceptable ranges, supporting the higher-order structure on the basis of converging evidence rather than any single index. Internal consistency was high [ω = .922, 95% CI (.915, .929); α = .863, 95% CI (.851, .875)], exceeding both the value reported by Gierk et al. [α = .81 (4)] and the pooled estimate from a recent meta-analysis [α = .80, 95% CI [.77, .83] (11)]. The pattern of latent correlations was consistent with the construct validity of the SSS-8 and with the well-established overlap between somatic symptoms, depression, and anxiety (50). Substantial positive correlations with depression (r = .80) and anxiety (r = .76), and negative correlation with well-being (r = -.67) supported validity. The latent correlations with depression and anxiety were higher than the manifest correlations reported by Gierk et al. [PHQ-2: r = .57; GAD-2: r = .55 (4)], which is expected given that latent correlations correct for measurement error. The finding that SSS-8 scores predicted health care utilization even after controlling for depression and anxiety provides further evidence that the SSS-8 captures a distinct dimension of symptom burden. The IRR of 1.097 per point is slightly lower than the value reported by Gierk et al. [IRR = 1.12 (4),], which is expected given the shorter recall period for doctor visits in the present study (three months vs. twelve months). The stepped increase across severity categories underscores the clinical utility of the SSS-8 severity classification. Regarding clinical interpretation of change scores, the minimal clinically important difference (MCID) for the SSS-8 has been established at 3 points (11, 51), which provides a useful benchmark for evaluating treatment effects in clinical practice. Notably, the observed mean difference between 2012 and 2021 (1.71 points) falls below this threshold, indicating that while the increase in somatic symptom burden was statistically significant (d = -0.40), it does not exceed the MCID at the individual level. Nevertheless, the significant shift in severity category distributions underscores that even changes below the individual-level MCID can be meaningful at the population level.

The overall factor structure of the SSS-8 was invariant across gender, age, gender × age, and clinical status. Strict invariance was supported for depression and anxiety. For gender, age, and gender × age, full scalar invariance was not achieved; however, partial scalar invariance was established after freeing the intercept of Item 4 (headaches). This finding is consistent with the well-documented gender and age differences in headache prevalence (52, 53) and suggests that while the overall measurement properties of the SSS-8 are equivalent across groups, the headache item functions slightly differently across gender and age groups. Importantly, partial scalar invariance is generally considered sufficient for meaningful group comparisons (54). The consistent pattern of Item 4 (headaches) showing the lowest item-total correlation (49), the lowest factor loading (57), and differential item functioning across gender and age converges with the meta-analytic finding that domain-specific factors contribute substantially to SSS-8 response patterns (11). The headache item appears to tap into a symptom domain with particularly pronounced demographic variation, which should be considered when interpreting group differences. This finding extends previous research on the SSS-8 and is consistent with the measurement invariance results reported for related instruments, including the PHQ-9 (14) and the GAD-7 (16).

Women reported higher somatic symptom levels than men, consistent with the existing literature (55, 56). The largest gender difference was observed for headaches (δ = -0.24), which is consistent with epidemiological data on the higher prevalence of headaches in women (53, 54, 58). Back pain and limb pain showed negligible gender differences. The cross-cohort comparison between the present sample and the original normative sample collected by Gierk et al. (4) in 2012 revealed a significant increase in somatic symptom burden over the nine-year interval (d = -0.40, 95% CI [-0.45, -0.34]). This increase was observed across all eight items and was more pronounced in women (d = -0.44) than in men (d = -0.37). The proportion of participants in the “no to minimal” severity category decreased from 67.8% to 53.8%, while the proportions in all higher categories increased. These findings are consistent with recent evidence showing that population norms for common mental health indicators are not static over time, as reflected in an upward shift of PHQ-9 norms (14) and in broader temporal variation in anxiety symptom levels, underscoring the need for regular updates of population norms.

### 4.1 Limitations

Several limitations should be noted. First, the response rate of 42.6% is lower than the 57% response rate in the original validation study (4). However, general population studies commonly have significantly lower response rates than clinical studies and the response rate of this study is comparable to similar surveys (58). Second, data analysis was carried out on one imputed data set. Future studies should consider multiple imputation with pooling. Third, the cross-sectional design does not permit causal conclusions regarding the relationship between somatic symptoms and health care utilization. Fourth, the RMSEA values from the CFA models exceeded conventional thresholds in both M1 and M2. While RMSEA is known to be inflated for short scales with large samples under WLSMV estimation (46), this does not by itself resolve the elevated values, and the model evaluation in the present study therefore relied on the convergence of multiple fit indices rather than RMSEA alone. Fifth, the recall period for health care utilization (three months) differed from the original study (twelve months), which should be considered when comparing IRR values across studies. Sixth, the cross-sectional design precludes the assessment of test-retest reliability. In this context, future research should examine the temporal stability of the SSS-8 factor structure using longitudinal measurement invariance in panel data. Brauer and Proyer (59) recently demonstrated this approach for the PHQ-4 using multiple waves of the German Socioeconomic Panel Innovation Sample (SOEP-IS), examining longitudinal measurement invariance and time-lagged correlations. Applying a similar design to the SSS-8 would provide evidence on whether the measurement properties remain stable over time and would support the inclusion of the SSS-8 in further waves of established panel studies. A recent meta-analysis identified only two studies evaluating this property for the SSS-8 (11), highlighting an important area for future research. Seventh, the survey was conducted during the COVID-19 pandemic (post-lockdown, during the vaccination campaign in 2021), which may have influenced somatic symptom levels and health care seeking behavior. It remains unclear whether the observed increase in symptom burden reflects a temporary deviation or a sustained shift.

### 4.2 Conclusion

The present study provides evidence for the reliability, factorial validity, construct validity, and measurement invariance of the SSS-8 in a representative German community sample. Updated population norms are provided for clinical practice. These percentiles are tabulated (see **Table 3**; **Supplementary Data Sheet 1**) for different age ranges and available both gender-specific and gender-unspecific. The SSS-8 severity categories were associated with a stepped increase in health care utilization, supporting their clinical utility. The cross-cohort comparison revealed a significant increase in somatic symptom burden over the past decade, underscoring the necessity of updating population norms at regular intervals.

## Data Availability

The datasets presented in this article are not readily available because ethics board approval did not include open data sharing. Questions concerning the data should be addressed to EB. Requests to access the datasets should be directed to elmar.braehler@medizin.uni-leipzig.de.
