# Supplementary material for: Psychometric evaluation and community norms of the Somatic Symptom Scale-8 based on a representative German sample
Source: Front Psychiatry. 2026 May 22;17:1851921. doi: 10.3389/fpsyt.2026.1851921 (PMC13236644; doi:10.3389/fpsyt.2026.1851921)
Supplement: Supplementary file 2 [file Table1.docx]

Supplementary Material

## Supplementary Tables

eTable 1. Gender differences in the SSS-8 total score and individual item scores

|  | Sample of Men (*N* = 1,217) | | | | Sample of Women (*N* = 1,297) | | | | Group Differences | | |
| --- | --- | --- | --- | --- | --- | --- | --- | --- | --- | --- | --- |
|  | *M* | *SD* | *Skew* | *Kurt* | *M* | *SD* | *Skew* | *Kurt* | *p* | ES (δ/d) | 95% CI |
| Stomach or bowel problems | 0.35 | 0.66 | 2 | 3.61 | 0.56 | 0.78 | 1.28 | 0.95 | < .001 | δ = -0.15 | [-0.18, -0.11] |
| Back pain | 0.91 | 0.98 | 0.8 | -0.25 | 0.90 | 0.97 | 0.88 | 0.06 | .707 | δ = 0.01 | [-0.04, 0.05] |
| Pain in arms/legs/joints | 0.65 | 0.94 | 1.35 | 0.98 | 0.67 | 0.95 | 1.31 | 0.87 | .607 | δ = -0.01 | [-0.05, 0.03] |
| Headaches | 0.53 | 0.73 | 1.37 | 1.6 | 0.87 | 0.83 | 0.8 | 0.43 | < .001 | δ = -0.24 | [-0.27, -0.19] |
| Chest pain/ shortness of breath | 0.22 | 0.59 | 3.13 | 10.05 | 0.25 | 0.64 | 2.94 | 9.03 | .173 | δ = -0.02 | [-0.05, 0.01] |
| Dizziness | 0.21 | 0.56 | 3.24 | 12.23 | 0.31 | 0.70 | 2.6 | 7.06 | < .001 | δ = -0.06 | [-0.09, -0.03] |
| Feeling tired/ low energy | 0.66 | 0.94 | 1.43 | 1.41 | 0.83 | 1.03 | 1.1 | 0.29 | < .001 | δ = -0.09 | [-0.13, -0.05] |
| Trouble sleeping | 0.55 | 0.91 | 1.74 | 2.45 | 0.73 | 0.99 | 1.32 | 1.01 | < .001 | δ = -0.11 | [-0.15, -0.07] |
| SSS-8 Total | 4.10 | 4.61 | 1.71 | 3.16 | 5.14 | 5.03 | 1.42 | 2.2 | < .001 | d = -0.22 | [-0.29, -0.14] |

*Note.* M = Mean; SD = Standard Deviation; Skew = skewness; Kurt = kurtosis; Cliff’s δ (ordinal); Total: Cohen’s d (quasi-metric, SSS-8 total score). Negative values = higher scores in females. P from permutation tests based on 10,000 resamples*.* One participant identified as diverse, included in the Total Sample but excluded from gender-stratified analyses.

eTable 2. Results of measurement invariance analyses regarding age, gender, anxiety (GAD-2), and depression (PHQ-2)

| Group | χ² | df | CFI | ΔCFI | RMSEA | RMSEA 90% CI | ΔRMSEA | Decision |
| --- | --- | --- | --- | --- | --- | --- | --- | --- |
| **Gender (male, female)** |  |  |  |  |  |  |  |  |
| Configural | 352.6 | 34 | 0.981 | — | 0.086 | [0.078, 0.095] | — | — |
| Threshold | 335.6 | 36 | 0.982 | +0.001 | 0.081 | [0.074, 0.089] | -0.005 | ✓ |
| Metric | 311.3 | 41 | 0.984 | +0.002 | 0.072 | [0.065, 0.080] | -0.009 | ✓ |
| Scalar | 498.9 | 49 | 0.973 | -0.011 | 0.085 | [0.079, 0.092] | +0.013 | ✗ |
| Partial scalar | 375.6 | 48 | 0.981 | -0.003 | 0.074 | [0.067, 0.081] | +0.002 | ✓p |
| Partial strict | 373.5 | 56 | 0.981 | +0.000 | 0.067 | [0.061, 0.074] | -0.007 | ✓p |
| **Age** |  |  |  |  |  |  |  |  |
| Configural | 354.9 | 34 | 0.978 | — | 0.087 | [0.079, 0.095] | — | — |
| Threshold | 338.2 | 36 | 0.979 | +0.001 | 0.082 | [0.074, 0.090] | -0.005 | ✓ |
| Metric | 316.2 | 41 | 0.981 | +0.002 | 0.073 | [0.066, 0.081] | -0.009 | ✓ |
| Scalar | 690.9 | 49 | 0.956 | -0.025 | 0.102 | [0.095, 0.109] | +0.029 | ✗ |
| Partial scalar | 397.6 | 48 | 0.976 | -0.005 | 0.076 | [0.069, 0.083] | +0.003 | ✓p |
| Partial strict | 458.1 | 56 | 0.972 | -0.004 | 0.076 | [0.069, 0.082] | +0.000 | ✓p |
| **Gender × Age** |  |  |  |  |  |  |  |  |
| Configural | 357.3 | 68 | 0.980 | — | 0.082 | [0.074, 0.091] | — | — |
| Threshold | 359.5 | 80 | 0.981 | +0.001 | 0.075 | [0.067, 0.083] | -0.007 | ✓ |
| Metric | 364.4 | 95 | 0.982 | +0.001 | 0.067 | [0.060, 0.075] | -0.008 | ✓ |
| Scalar | 883.8 | 119 | 0.948 | -0.034 | 0.101 | [0.095, 0.108] | +0.034 | ✗ |
| Partial scalar | 523.1 | 116 | 0.972 | -0.010 | 0.075 | [0.068, 0.081] | +0.008 | ✓p |
| Partial strict | 610.1 | 140 | 0.968 | -0.004 | 0.073 | [0.067, 0.079] | -0.002 | ✓p |
| **Anxiety (no, yes)** |  |  |  |  |  |  |  |  |
| Configural | 348.0 | 34 | 0.975 | — | 0.086 | [0.078, 0.094] | — | — |
| Threshold | 344.3 | 36 | 0.975 | +0.000 | 0.083 | [0.075, 0.091] | -0.003 | ✓ |
| Metric | 297.7 | 41 | 0.979 | +0.004 | 0.071 | [0.063, 0.078] | -0.012 | ✓ |
| Scalar | 321.8 | 49 | 0.978 | -0.001 | 0.067 | [0.060, 0.074] | -0.004 | ✓ |
| Strict | 332.5 | 57 | 0.978 | +0.000 | 0.062 | [0.056, 0.069] | -0.005 | ✓ |
| **Depression (no, yes)** |  |  |  |  |  |  |  |  |
| Configural | 349.8 | 34 | 0.973 | — | 0.086 | [0.078, 0.094] | — | — |
| Threshold | 345.7 | 36 | 0.973 | +0.000 | 0.083 | [0.075, 0.091] | -0.003 | ✓ |
| Metric | 314.1 | 41 | 0.976 | +0.003 | 0.073 | [0.065, 0.080] | -0.010 | ✓ |
| Scalar | 315.1 | 49 | 0.977 | +0.001 | 0.066 | [0.059, 0.073] | -0.007 | ✓ |
| Strict | 318.2 | 57 | 0.978 | +0.001 | 0.060 | [0.054, 0.067] | -0.006 | ✓ |

*Note.* All fit statistics are scaled (WLSMV). CFI = Comparative Fit Index; ΔCFI = change in CFI; RMSEA = Root Mean Square Error of Approximation; ΔRMSEA = change in RMSEA. ✓= ΔCFI < -.010 complemented by ΔRMSEA ≥ .015 indicates a violation of measurement invariance; ✓ = measurement invariance for the respective level; ✓p = partial invariance (Item 4 intercept freed).

eTable 3. Latent and manifest correlations between the SSS-8 and depression symptoms (PHQ-2), anxiety symptoms (GAD-2), loneliness (UCLA Loneliness Scale-3), well-being (WHO-5).

| **Construct** | **r (latent)** | **95% CI** | **r (manifest)** | **95% CI** |
| --- | --- | --- | --- | --- |
| Depression (PHQ-2) | .81*** | [.77, .83] | .65*** | [.63, .67] |
| Anxiety (GAD-2) | .76*** | [.72, .79] | .60*** | [.57, .62] |
| Well-being (WHO-5) | -.67*** | [-.70, -.64] | -.60*** | [-.62, -.57] |
| Loneliness (UCLA-3) | .57*** | [.53, .61] | .49*** | [.46, .52] |

*Note.* Latent correlations estimated via SEM with WLSMV. 95% Cis from bootstrap with 1,000 replications. Manifest correlations: Pearson. All p < .001*.*

eTable 4. Distribution of SSS-8 Severity Categories: Cross-Cohort Comparison.

|  | **Total Sample** | | **Sample of Men** | | **Sample of Women** | |
| --- | --- | --- | --- | --- | --- | --- |
|  | 2012 n (%) | 2021 n (%) | 2012 n (%) | 2021 n (%) | 2012 n (%) | 2021 n (%) |
| No to minimal | 1,684 (67.8) | 1,353 (53.8) | 826 (71.3) | 717 (58.9) | 858 (64.7) | 636 (49.0) |
| Low | 560 (22.5) | 618 (24.6) | 241 (20.8) | 282 (23.2) | 319 (24.1) | 335 (25.8) |
| Medium | 158 (6.4) | 296 (11.8) | 62 (5.4) | 124 (10.2) | 96 (7.2) | 172 (13.3) |
| High | 50 (2.0) | 140 (5.6) | 16 (1.4) | 51 (4.2) | 34 (2.6) | 89 (6.9) |
| Very high | 32 (1.3) | 108 (4.3) | 13 (1.1) | 43 (3.5) | 19 (1.4) | 65 (5.0) |
| *Group Differences* | *χ²(4) = 164.58, < .001* | | *χ²(4) = 64.51, < .001* | | *χ²(4) = 104.41, < .001* | |

*Note.* Severity categories: no to minimal (0-3), low (4-7), medium (8-11), high (12-15), very high (16-32) (4).

eTable 5. Inter-Item Correlations of the SSS-8 (*N* = 2,515).

| **Item** | **M** | **SD** | **1** | **2** | **3** | **4** | **5** | **6** | **7** |
| --- | --- | --- | --- | --- | --- | --- | --- | --- | --- |
| Stomach or bowel problems | 0.46 | 0.73 | — |  |  |  |  |  |  |
| Back pain | 0.91 | 0.98 | .41 [.37, .44] | — |  |  |  |  |  |
| Pain in arms/legs/joints | 0.67 | 0.95 | .37 [.34, .40] | .64 [.61, .66] | — |  |  |  |  |
| Headaches | 0.71 | 0.81 | .38 [.35, .41] | .33 [.30, .37] | .29 [.25, .33] | — |  |  |  |
| Chest pain/ shortness of breath | 0.23 | 0.61 | .39 [.35, .42] | .37 [.34, .40] | .45 [.41, .48] | .35 [.31, .38] | — |  |  |
| Dizziness | 0.27 | 0.65 | .38 [.35, .41] | .36 [.32, .39] | .45 [.42, .48] | .37 [.34, .41] | .61 [.58, .63] | — |  |
| Feeling tired/ low energy | 0.75 | 1.00 | .45 [.42, .48] | .49 [.46, .52] | .56 [.53, .59] | .43 [.40, .46] | .52 [.49, .55] | .53 [.50, .56] | — |
| Trouble sleeping | 0.65 | 0.96 | .43 [.40, .47] | .47 [.44, .50] | .49 [.46, .52] | .40 [.36, .43] | .47 [.44, .50] | .52 [.49, .55] | .68 [.66, .70] |

*Note.* Pearson correlations with 95% confidence intervals in brackets. All correlations p < .01

## Supplementary Figures

eFigure 1. MGCFA models for measurement invariance Analysis


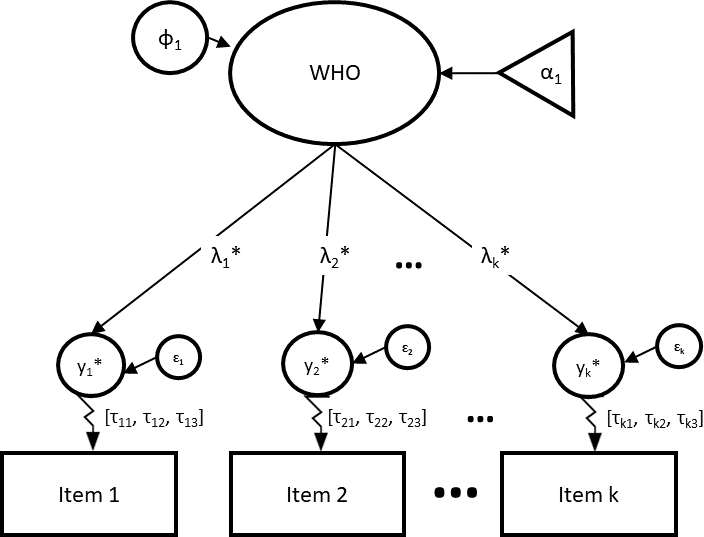


*Note.*

(1) Configural invariance: Same CFA model fit in all groups;

(2) Threshold Invariance: thresholds τ1i…τ4i constrained to be equal across groups;

(3) Weak Invariance: additionally, factor loadings λi constrained to be equal across groups;

(4) Strong Invariance: additionally, intercepts constrained to be equal across groups;

(5) Full Invariance: additionally, residual variances ϵi constrained to be equal across groups.

eFigure 2. CFA Path Diagram: Unidimensional Model (M1).

*Note.* N = 2,515. WLSMV estimation.

eFigure 3. CFA Path Diagram: Higher-Order Model (M2)

Note. Path diagram of the higher-order confirmatory factor analysis model (M2). Circles represent latent variables; rectangles represent observed items. Single-headed arrows indicate factor loadings (standardized estimates shown on paths). The second-order general factor (Somatic Symptom Burden) explains the covariance among three first-order domain factors (Pain, Cardiopulmonary, Fatigue). Item 1 (stomach or bowel problems; gastrointestinal domain) loads directly on the second-order factor because specifying a single-item first-order factor resulted in a non-invertible information matrix under WLSMV estimation. Model fit: CFI = .979, TLI = .966, RMSEA = .091 (90% CI [.083, .099]), SRMR = .046.
